# Demographic, occupational factors and pandemic-related stressors associated with heightened mental health difficulties among UK health and social care workers supported by regional Resilience Hubs during the COVID-19 pandemic

**DOI:** 10.1136/bmjopen-2023-082817

**Published:** 2025-02-25

**Authors:** Filippo Varese, Kate Allsopp, Lesley-Anne Carter, Jack Wilkinson, Gemma E Shields, Aleix Rowlandson, Priscilla Chung, Alysha A Hassan, Hannah White, Sally-Anne Wright, Ellie Young, Jess Davey, Alan Barrett, Gita Bhutani, Daniel Hind, Katherine McGuirk, Fay Huntley, May Sarsam, Holly Walker, Joanne Jordan, Hein Ten Cate, Ruth Watson, Jenni Willbourn, Paul French

**Affiliations:** 1 School of Health Sciences, Manchester Academic Health Science Centre, The University of Manchester, Manchester, UK; 2 Complex Trauma & Resilience Research Unit, Greater Manchester Mental Health NHS Foundation Trust, Manchester, UK; 3 Centre for Biostatistics, Division of Population Health, Health Services Research and Primary Care, The University of Manchester, Manchester, UK; 4 Centre for Health Economics, University of Manchester, Manchester, UK; 5 Lancashire and South Cumbria Resilience Hub, Lancashire and South Cumbria NHS Foundation Trust, Preston, UK; 6 Cheshire & Merseyside Resilience Hub, Mersey Care NHS Foundation Trust, Liverpool, UK; 7 Greater Manchester Resilience Hub, Pennine Care NHS Foundation Trust, Ashton-under-Lyne, UK; 8 Humber & North Yorkshire Resilience Hub, Tees Esk and Wear Valleys NHS Foundation Trust, Darlington, UK; 9 School of Health & Society, University of Salford, Salford, UK; 10 ScHARR, The University of Sheffield, Sheffield, UK; 11 NHS Greater Manchester Health and Social Care Partnership, Manchester, UK; 12 Doctorate of Clinical Psychology, University of Edinburgh, Edinburgh, UK; 13 Greater Manchester Mental Health NHS Foundation Trust, Manchester, UK; 14 Manchester Metropolitan University, Manchester, UK; 15 Pennine Care NHS Foundation Trust, Ashton-under-Lyne, UK

**Keywords:** MENTAL HEALTH, COVID-19, Occupational Stress, Psychometrics

## Abstract

**Abstract:**

**Background:**

During the COVID-19 pandemic, 40 mental health and well-being hubs were funded in England to support health and social care staff affected by the pandemic.

**Aims:**

To describe the characteristics of staff accessing four hubs for support and identify characteristics associated with clinically significant mental health difficulties and work and social functioning.

**Method:**

Routinely collected screening data were analysed from 1973 individuals across 4 hubs, including mental health, demographic and occupational data and pandemic-related stressors. Factors associated with clinically significant mental health difficulties were identified via logistic regression.

**Results:**

Most hub clients identified as white women who worked for the UK National Health Service; other groups were less well represented. Hub clients reported high levels of clinically significant mental health difficulties: 60% had severe and often co-occurring difficulties (ie, depression, anxiety, post-traumatic stress disorder or alcohol use) and 80% reported significantly impaired functioning. Younger age, disability status, identifying as from a minority ethnic group, and sexual orientations excluding heterosexual were associated with higher likelihood of having clinically significant mental health difficulties. Suffering financial loss during the pandemic, and prepandemic emotional well-being concerns were the most consistent factors associated with higher difficulties.

**Conclusions:**

The hubs supported health and social care staff with significant mental health difficulties. Outreach and engagement with under-represented groups should be undertaken to address potential barriers to service access. The findings add to the knowledge base on the support needs of the health and social care workforce and the planning of support in response to future crises.

**STRENGTHS AND LIMITATIONS OF THIS STUDY**

- This is the first study exploring the characteristics of health and social care staff registering for support with staff well-being hubs (‘Resilience Hubs’) funded by National Health Service (NHS) England during the COVID-19 pandemic, and which of these characteristics were associated with more severe mental health difficulties.
- The study has a large sample size of 1973 individuals who gave consent for the use of their data for research purposes, across four Resilience Hubs, representing 83% of the staff who referred themselves to the hubs between 1 June 2020 and 31 December 2021.
- The study is limited by the lack of a comparison group, for example, staff who accessed alternative support services in a region without hub support available.
- Finally, the current study explores 10% of the 40 hubs set up during the pandemic. NHS England guidance for the setup of staff well-being hubs was broad and has been operationalised with high levels of local variation across hubs; therefore, these findings may not be representative of all staff well-being hubs.

## Introduction

 The COVID-19 pandemic has affected the mental health of health and social care staff.^1 2^ Systematic reviews have demonstrated high levels of depression, anxiety and post-traumatic stress symptoms throughout the pandemic.^3 4^ A pooled prevalence from one review suggested that, globally, 49% of healthcare staff reported problems with insomnia, 47% anxiety and 37% with post-traumatic stress.^5^ Research suggests that the mental health of staff from black, Asian and minority ethnic communities may have been particularly affected.^6^ Staff working in intensive care units (ICUs) or critical care services are more likely to have experienced post-traumatic stress and other mental health difficulties.^7^ While there is limited research on the mental health of care home staff, the impact appears no less severe.^8^

National Health Service (NHS) England, the commissioning body that oversees the publicly funded healthcare system, the National Health Service (NHS), in England, funded 40 resilience or well-being hubs to support staff during the pandemic.^9^ These hubs were modelled on a service called the Greater Manchester Resilience Hub, which was originally set up to support people affected by a 2017 terrorist bombing in Manchester (UK). These resilience/well-being hubs offered a range of services to support health and social care staff affected by their work during the pandemic. Details of the support offered by the four Resilience Hubs involved in this study are published elsewhere.^10^ The purpose of these services during the pandemic was to facilitate access to NHS-recommended mental health support for health and social care professionals. These dedicated services, which had a focus on proactive outreach, were established with the aim of resolving known barriers to help-seeking among health and social care staff, and to avoid placing additional strain on other mental health services during the pandemic.^11^ Support offered by the hubs is consistent with NHS guidance on supporting mental well-being at work.^12^

As newly established services, the characteristics of people accessing hub services during the pandemic, or how these characteristics related to mental health needs, were not known. Information regarding the characteristics of staff who presented with more severe mental health issues (ie, potentially requiring more intensive and bespoke mental health support) gathered during the pandemic may be beneficial to the more efficient planning of support services such as the Resilience Hubs should their activation be required in response to novel crises, and/or ongoing staff mental health support initiatives. The objectives of this study were therefore to analyse the demographic and occupational characteristics of health and social care staff who accessed four Resilience Hubs for support during the pandemic, to explore characteristics that were associated with higher mental health needs and work and social functional difficulties, to identify health and social care staff who may benefit from mental health support.

The quantitative findings presented in this paper are one component of the wider mixed methods evaluation to maximise learning from the UK’s response to the early phases of the COVID-19 pandemic in relation to the implementation of this innovative system of support for responding to increased mental health needs populations and specific groups affected large scale crises.^13^ Findings pertaining to other workstreams of the Resilience Hubs Evaluation are reported elsewhere.^10 14^

## Method

### Setting

Four hubs were involved in the study. Hub names have been anonymised and are described below as sites A–D. The hubs became operational at different time points due to variations in setup times, and most of the hubs involved in the study opened in stages to different staffing groups. The earliest hub to open was site D in May 2020. The other hubs became operational between November 2020 and February 2021. Mental health screening formed a part of the self-referral process at all hubs involved in the evaluation, although there were some variations across services. All hubs encouraged online self-referral, and the completion of mental health screening data was either conducted as part of the online self-referral form, or, at one hub, online questionnaires were sent to hub clients after their referral was accepted and prior to their first assessment session. Further information regarding what these mental health services comprised, and how people could refer themselves, can be found in a detailed service mapping published elsewhere.

### Participants

Hub clients were defined as staff members eligible for hub support who had been referred or self-referred for individual support from one of four Resilience Hubs in the North West of England. There were no exclusion criteria. Health and social care staff supported by the hubs included, broadly, staff working with NHS or private healthcare organisations, and staff working in social care organisations, such as residential care homes. Clinical, managerial, administrative and ancillary staff at in-scope organisations were all eligible to access hubs for support. Some further information is provided elsewhere about in-scope staffing groups for hubs involved in this study.^10^

To avoid confusion between staff working at the hubs and people accessing hub services, the paper will refer to the latter as ‘hub clients’ or ‘participants’ for hub clients who were included in the research. All participants (1) were over 18 years of age, (2) completed screening at one of the Hubs between 1 June 2020 and 31 December 2021 and (3) consented for their data to be used for research purposes.

### Data sources

All hubs routinely collected data on symptoms of depression (using the Patient Health Questionnaire; PHQ-9),^15^ anxiety (the General Anxiety Disorder scale; GAD-7),^16^ and social and occupational functional impairment (the Work and Social Adjustment Scale^17^; WSAS). The hubs also administered screening tools for post-traumatic stress disorder (PTSD), but different instruments were employed at the participating hubs (sites A and B used the PTSD Checklist for the DSM-5, PCL-5)^18^; sites C and D used the International Trauma Questionnaire, ITQ.^19^ Three hubs (sites A, C and D) also collected data on harmful alcohol use using the Alcohol Use Disorders Identification Test (AUDIT).^20^

Details on the scoring of the above instruments, including the scoring thresholds and criteria we used to examine the prevalence of clinically significant difficulties in the above domains (ie, depression, anxiety, post-traumatic stress, problematic alcohol use and functioning), are summarised in table 1. Hereafter, the term ‘caseness’ is used to refer to meeting these thresholds for clinically significant difficulties.

**Table 1:** Scoring of the routine self-report mental health (MH) screening measures administered to Hub clients at the four participating sites

Domain	Measure	Thresholds to evaluate the severity of MH difficulties	Availability of the measure at the four sites	‘Caseness’ definition for the current regression analyses
Depression	PHQ-9	Severe depression=20–29Moderately severe depression=15–19Moderate depression=10–14Mild depression=5–9No depression=0–4	Hubs A, B, C and D	Scores suggestive of at least moderate depression (PHQ ≥ 10).
Anxiety	GAD-7	Severe anxiety=15–21Moderate anxiety=10–14Mild anxiety=5–9Minimal anxiety=0–4	Hubs A, B, C and D	Scores suggestive of at least moderate anxiety (GAD-7 ≥ 10).
Post-traumatic stress	PCL-5	Probable PTSD=31–80Subthreshold for PTSD=0–30	Hubs A and B	Scores suggestive of probable PTSD (PCL-5 ≥ 31).
	ITQ	Probable PTSD=scores of 2+on at least one symptom/item of each PTSD cluster (intrusions, avoidance, hyperarousal); plus scores of 2+ on at least one item assessing associated functional impairmentProbable cPTSD=Meeting criteria for probable PTSD above; plus scores of 2+ on at least one symptom/item of each ‘disturbances of self organisation’ cluster (affect dysregulation, negative self-concept, disturbances in relationships); plus scores of 2+ on at least one item assessing associated functional impairmentSubthreshold for PTSD/cPTSD=Not meeting criteria for probable PTSD above	Hubs C and D	Meeting ITQ criteria for probable PTSD or CPTSD.
Problematic alcohol use	AUDIT	Possible alcohol dependence=20–40Harmful alcohol consumption=16–19Hazardous alcohol consumption=8–15Low-risk consumption=1–7	Hubs A, C and D	Scores suggestive of at least hazardous alcohol consumption (AUDIT ≥ 8).
Social and occupational impairment	WSAS	Moderately severe or worse impairment: 20–40Significant impairment=10–19Low/no impairment=0–9	Hubs A, B, C and D	Scores suggestive of at least significant functional impairment (WSAS ≥ 10)

AUDIT, Alcohol Use Disorders Identification Test; GAD7, General Anxiety Disorder scale; ITQ, International Trauma Questionnaire; PCL-5, PTSD Checklist for the DSM-5; PHQ-9, Patient Health Questionnaire; PTSD, post-traumatic stress disorder; WSAS, Work and Social Adjustment Scale.

The hubs also collected data on a range of hub clients’ self-reported characteristics relevant to the planned analyses, including (1) demographic data (age, gender, ethnicity, disability status and sexual orientation, (2) occupational and work environment characteristics (hub clients’ work setting and job role), (3) whether hub clients had prepandemic concerns about their emotional well-being/mental health (eg, ’Were you concerned about your emotional wellbeing/mental health before COVID-19?’) and (4) information on common impacts of COVID-19 during the acute phase of the pandemic. The latter covered whether the person had been impacted by COVID-19 in any of the following ways: (1) seconded to a different post; (2) moved to work in a different location; (3) undertaking new tasks within usual role; (4) been ill with confirmed COVID-19 (recovered at home); (5) been ill with confirmed COVID-19 (including being in hospital); (6) family member been ill with confirmed COVID-19 (recovered at home); (7) family member been ill with confirmed COVID-19 (included being in hospital); (8) experienced family/close friend bereavement and (9) suffered financial loss within the household.

### Procedures

All individuals screened by the hubs were invited to give consent for their anonymised data to be used for research purposes. This consent to data use was asked at one time point, as the screening questionnaires were completed at a single time point as part of the routinely collected data at the point of self-referral to hubs. However, hub clients could request hubs to withdraw this consent at any time. At the point of data lock for the study (31 December 2021), screening data were only transferred to the research team for hub clients who had consented to anonymised data use for research purposes on that date.

Relevant data for all consenting hub clients were extracted from the hubs’ electronic patient records systems, cleaned and anonymised by research assistants based at each Hub. The data were compiled onto a central database managed by the study statisticians, who performed quality checking and relevant recoding/cleaning ahead of the planned analyses.

## Analysis

For each hub, we numerically summarised data on participant demographic and occupational characteristics, reported COVID-19 impacts and prepandemic emotional well-being/mental health concerns. Data from mental health screening questionnaires were summarised numerically as total scores and used to determined the number of participants meeting threshold for clinically significant difficulties across the assessed domains. A series of logistic regression models, adjusted for hubs due to the multisite nature of the data, were conducted to examine the association between each independent variable and ‘caseness’ on each mental health screening outcome variable. To evaluate whether these relationships varied across the hubs, all models were refitted with an interaction between the variable under consideration and the site. The interaction was assessed using a likelihood ratio test for logistic regression models. To offer some protection against spurious findings arising from multiple testing, we used a significance threshold of p<0.001 for interaction analyses to identify potential differences across hubs. Owing to the large number of tests performed, p values should be considered nominal; significant associations are best interpreted as exploratory. A final set of analyses was conducted using proportional odds ordinal logistic regression analyses, adjusted for site, to identify potential factors associated with higher ‘overall severity’ variable across the various standardised screening measures collected by the hubs. This three-level categorical variable was defined by the highest severity categorisation received on any of hub’s screening questionnaires (further detail on the definition of this derived variable is available in full, see online supplemental material).

### Missing data

Due to different data collection policies at the participating hubs (ie, whether or not hub clients were invited to complete clinical screening measures at registration, and whether they were given the option of ‘skipping’ particular items or instruments), data availability varied according to site. For example, hub B presented notably higher missing data on the mental health screening measures that hub clients were invited to complete (ie, approximately 11%) compared with other hubs (where missing data was in most cases <1%). For most of the other variables considered in our analyses, data missingness was <3%, with the notable exclusion of certain demographic variables (in particular ethnicity and sexual orientation, which presented higher numbers of not stated and ‘prefer not to say’ answers at certain sites). As the above suggests that data were unlikely to be missing at random, only observed data were used in the descriptive and regression analyses reported below.

## Results

Data for 1973 hub clients across the four Resilience Hubs were included in the analyses, representing the 83% of people who referred themselves to the hubs between 1 June 2020 and 31 December 2021 and gave consent for the use of their anonymised data for research purposes.

In terms of occupational background, most hub clients were NHS employees. A sizeable minority of these NHS employees (30% of all NHS participants) worked in intensive care settings. Only a relatively small proportion reported working in social care settings (6%) or in emergency services (5%; see online supplemental Table 2 for a more detailed breakdown of the occupational characteristics of the sample). The demographic characteristics of the sample are displayed in table 2. Given the substantial preponderance of NHS employees among hub clients and the small representation of certain occupational sectors in the available data, subsequent analyses aimed at identifying occupational variables associated with greater mental health needs focused on more specific occupational variables that may covey heightened risk (eg, working in high-risk settings like ICUs/critical care) as opposed to broad occupational sectors.

**Table 2:** N (%) of the demographic characteristics of the included Hub clients

	Site A (n=475)	Site B(n=367)	Site C (n=400)	Site D(n=731)	Total(N=1973)
Mean age (SD)	40.6 (10.6)0% missing	38.8 (11.4)3.0% missing	42.3 (11.2)0% missing	41.9 (11.4)0% missing	41.1 (11.2)0.5% missing
Ethnicity					
White British	433 (91.4)	327 (91.6)	367 (92.4)	586 (88.5)	1713 (90.6)
Other white	12 (2.5)	13 (3.6)	11 (2.8)	29 (4.4)	65 (3.4)
Black	1 (0.2)	1 (0.2)	4 (1.0)	7 (1.1)	13 (0.7)
Asian	20 (4.2)	10 (2.8)	6 (1.5)	29 (4.4)	65 (3.4)
Mixed	6 (1.3)	4 (1.1)	6 (1.5)	8 (1.2)	24 (1.3)
Other	2 (0.4)	2 (0.6)	3 (0.8)	3 (0.5)	10 (0.5)
Missing/not stated	0.2% missing	2.7% missing	0.8% missing	9.4% missing	4.2% missing
Gender					
Woman	401 (84.4)	309 (86.3)	331 (82.8)	612 (84.2)	1653 (84.3)
Man	73 (15.4)	47 (13.1)	63 (15.8)	96 (13.2)	279 (14.2)
Identified in another way	1 (0.2)	2 (0.6)	2 (0.5)	19 (2.6)	24 (1.5)
Missing/not stated	0% missing	0% missing	1% missing	0.4% missing	0.6% missing
Sexual orientation					
Heterosexual	420 (90.1)	307 (89.0)	318 (94.6)	587 (92.3)	1632 (91.5)
Identified in another way	46 (9.9)	38 (11.0)	18 (5.4)	49 (7.8)	151 (8.5)
Prefer not to say/left blank	1.3% missing	6.0% missing	16.0% missing	13.0% missing	9% missing
Disability status (yes)	64 (13.5)	30 (8.2)	72 (18.0)	29 (4.0)	195 (10.9)

Overall, the demographic characteristics of hub clients were similar across hubs. The average age of clients was 41.1 years (SD=11.2), ranging from 38.8 years to 42.3 years across hubs. The available ethnicity data indicated that clients were predominantly of white British background (90% across hubs). In terms of gender and sexual orientation, 84% of hub clients identified as women, and 91.5% identified as straight/heterosexual. Self-reported information on disability status was more variable, ranging between 4% and 18% across hubs. Of note, these differences may due to variances in how questions on disability status were framed at different hubs (ie, at sites B and D items to confirm lack of a disability were embedded within an extensive, alphabetically ordered list of potential disabilities, which may have led to high levels of missingness).

As summarised in table 3, considerable proportions of participants experienced a range of adverse pandemic-related personal and occupational circumstances prior to completing the screening offer of the hubs, and many clients reported having emotional well-being concerns that preceded the onset of the pandemic.

**Table 3:** N (%) for of respondents endorsing COVID-19 impact items and pre-pandemic mental health/ emotional well-being concerns

Question	Site A (n=475)	Site B (n=367)	Site C (n=400)	Site D(n=731)	Total (n=1973)
Have you been impacted in any of these ways by COVID-19?					
Ill with COVID-19 (recovered at home)	147 (30.9)0% missing	84 (23.2)1.4% missing	144 (36.8)2.3% missing	204 (28.7)2.9% missing	580 (29.9) 1.5% missing
Ill with COVID-19 (including being in hospital)	19 (4.0)0% missing	10 (2.8)1.4% missing	23 (6.0)4.8% missing	12 (1.7)5.2% missing	64 (3.3)2.9% missing
Family member ill with COVID (recovered at home)	119 (25.0)0% missing	68 (18.8)1.4% missing	136 (35.0)2.8% missing	187 (26.77)4.2% missing	511 (26.5) 2.1% missing
Family member ill with COVID (including being in hospital)	37 (7.8)0% missing	14 (3.9)1.4% missing	39 (10.1)3.8% missing	60 (8.7)5.3% missing	150 (7.8) 2.7% missing
Suffered financial loss within the household	84 (17.7)0% missing	33 (9.1)1.4% missing	84 (21.4)2.0% missing	152 (21.5)3.3% missing	353 (18.2) 1.6% missing
Undertaking new tasks within usual role	245 (51.63)0% missing	173 (47.8)1.4% missing	193 (49.1)1.8% missing	409 (58.3)4.1% missing	1021 (52.7) 1.9% missing
Seconded or redeployed to a different post	116 (26.2)6.9% missing	46 (12.7)1.4% missing	48 (12.2)1.8% missing	109 (16.2)8.1% missing	319 (17.0) 5.2% missing
Moved to a different work location	153 (34)5.3% missing	61 (16.9)1.4% missing	105 (26.7)1.8% missing	253 (36.4)4.9% missing	572 (30.1) 3.7% missing
Bereavement	71 (14.9)0% missing	44 (12.2)1.4% missing	65 (17.1)4.8% missing	168 (23.8)3.3% missing	348 (18.0) 2.2% missing
Were you concerned about your emotional well-being before COVID?					
Yes	170 (36.3)	169 (46.9)	136 (34.0)	276 (38.3)	754 (38.6)
Unsure	102 (21.8)	57 (15.8)	64 (16.0)	124 (17.2)	347 (17.8)
	0% missing	1.9% missing	0% missing	1.5% missing	1.0% missing

### Mental health and functional screening data

As illustrated in table 4, a large proportion of hub clients had been negatively affected by significant mental health and/or functional difficulties. The proportion of participants presenting PHQ-9 scores above the cut-off for moderate depression was 81%. In terms of anxiety, 60% of participants had GAD-7 scores above the cut-off for moderate anxiety. In hubs that used the PCL-5, 59% of clients had scores suggestive of probable PTSD. Conversely, a lower observed prevalence of possible trauma-related disorders (PTSD and complex PTSD) was observed when the ITQ was used (34% at site C and 28% at site D). The proportion of participants presenting AUDIT scores above the cut-off for hazardous alcohol use was 23%. Most hub clients presented WSAS scores above threshold for significant impairment in functioning (79%).

**Table 4:** Mean (SD) and number (%) of participants meeting cut-offs for clinically significant difficulties across Hub screening measures

	Site A(n=475)	Site B(n=367)	Site C(n=400)	Site D(n=731)	Total (n=1973)
PHQ-9	14.4 (5.5)	13.8 (5.9)	13.2 (5.9)	11.4 (6.3)	12.9 (6.1)
None	21 (4.4)	15 (4.6)	27 (6.8)	117 (16.0)	180 (9.3)
Mild	73 (15.4)	76 (23.3)	94 (23.6)	185 (25.3)	428 (22.2)
Moderate	141 (29.7)	94 (28.9)	117 (29.3)	186 (25.4)	538 (27.9)
Moderately severe	149 (31.4)	78 (23.9)	94 (23.6)	159 (21.8)	480 (24.9)
Severe	91 (19.2)	63 (19.3)	67 (16.8)	84 (11.5)	305 (15.8)
*Missing*	0% missing	11.1% missing	0% missing	0% missing	2.1% missing
GAD-7	12.3 (4.9)	12.6 (5.4)	16 (5.5)	10.2 (6.1)	11.4 (5.7)
None	28 (5.9)	17 (5.2)	44 (11.0)	153 (20.9)	242 (12.5)
Mild	121 (25.5)	91 (28.0)	102 (25.6)	207 (28.3)	521 (27.0)
Moderate	146 (30.7)	84 (25.8)	124 (31.1)	164 (22.4)	518 (26.8)
Severe	180 (37.9)	133 (40.9)	129 (32.3)	207 (28.3)	649 (33.6)
Missing	0% missing	11.4% missing	0.3% missing	0% missing	2.2% missing
PCL-5	36.6 (16.6)	34.3 (16.7)	–	–	35.6 (16.7)
PTSD present	293 (61.7)	180 (55.4)			473 (59.1)
Missing	1.0% missing	11.4% missing	–	–	5.0% missing
ITQ score	–	–	8.8 (6.3)	8.2 (6.5)	8.4 (6.4)
PTSD present			40 (10.0)	56 (7.7)	96 (8.5)
Missing		–	0.3% missing	0% missing	0.1% missing
CPTSD present	–	–	97 (24.5)	147 (20.4)	244 (21.6)
Missing			1.0% missing	1.6% missing	1.4% missing
AUDIT	5.7 (5.8)	–	5.0 (5.1)	5.2 (5.0)	5.3 (5.3)
Low risk	351 (73.9)		322 (80.5)	564 (77.2)	1237 (77.0)
Hazardous	88 (18.5)		63 (15.8)	131 (17.9)	282 (17.6)
Harmful	18 (3.8)		5 (1.3)	23 (3.1)	46 (2.9)
Possible dependence	18 (3.8)		10 (2.5)	13 (1.8)	41 (2.6)
Missing	0% missing	–	0% missing	0% missing	0% missing
WSAS	18.9 (8.3)	17.5 (7.9)	17.9 (9.5)	15.1 (9.3)	17.0 (9.0)
Subclinical	65 (13.7)	55 (16.9)	77 (19.3)	213 (29.1)	410 (21.2)
Significant	213 (44.8)	152 (46.6)	170 (42.5)	311 (42.5)	846 (43.8)
Moderately severe or worse	197 (41.5)	119 (36.5)	153 (38.3)	207 (28.3)	676 (35.0)
Missing	0% missing	11.2% missing	0% missing	0% missing	2.1% missing
Overall severity					
Low	24 (5)	23 (6.3)	29 (7.3)	128 (17.5)	204 (10.3)
Moderate	104 (21.9)	71 (19.3)	128 (32.0)	230 (31.5)	533 (27.0)
High	347 (73.1)	232 (63.2)	243 (60.8)	373 (51.0)	1195 (60.6)
Missing	0% missing	11.2% missing	0% missing	0% missing	2.1% missing

Clinical cut-off scores for Hub screening measures: PHQ-9: 0–4=none, 5–9=mild, 10–14=moderate, 15–19=moderately severe, 20–29=severe; GAD-7: 0–4=none, 5–9=mild, 10–14=moderate, 15–21=severe; PCL-5: 31+probable PTSD; ITQ: probable PTSD diagnosis indicated by a score of 2+ on at least one symptom of each PTSD cluster along with associated functional impairment, probable cPTSD diagnosis indicated by meeting PTSD criteria and a score of 2+ on at least one symptom from each DSO cluster along with associated functional impairment; AUDIT: 1–7=low risk, 8–15=hazardous, 16–19=harmful, 20+=possible dependence; WSAS: 0–9=subclinical, 10–19=significant, 20+=moderately severe or worse.

AUDIT, Alcohol Use Disorders Identification Test; GAD-7, General Anxiety Disorder scale; ITQ, International Trauma Questionnaire; PCL-5, PTSD Checklist for the DSM-5; PHQ-9, Patient Health Questionnaire; WSAS, Work and Social Adjustment Scale.

**Figure 1:**
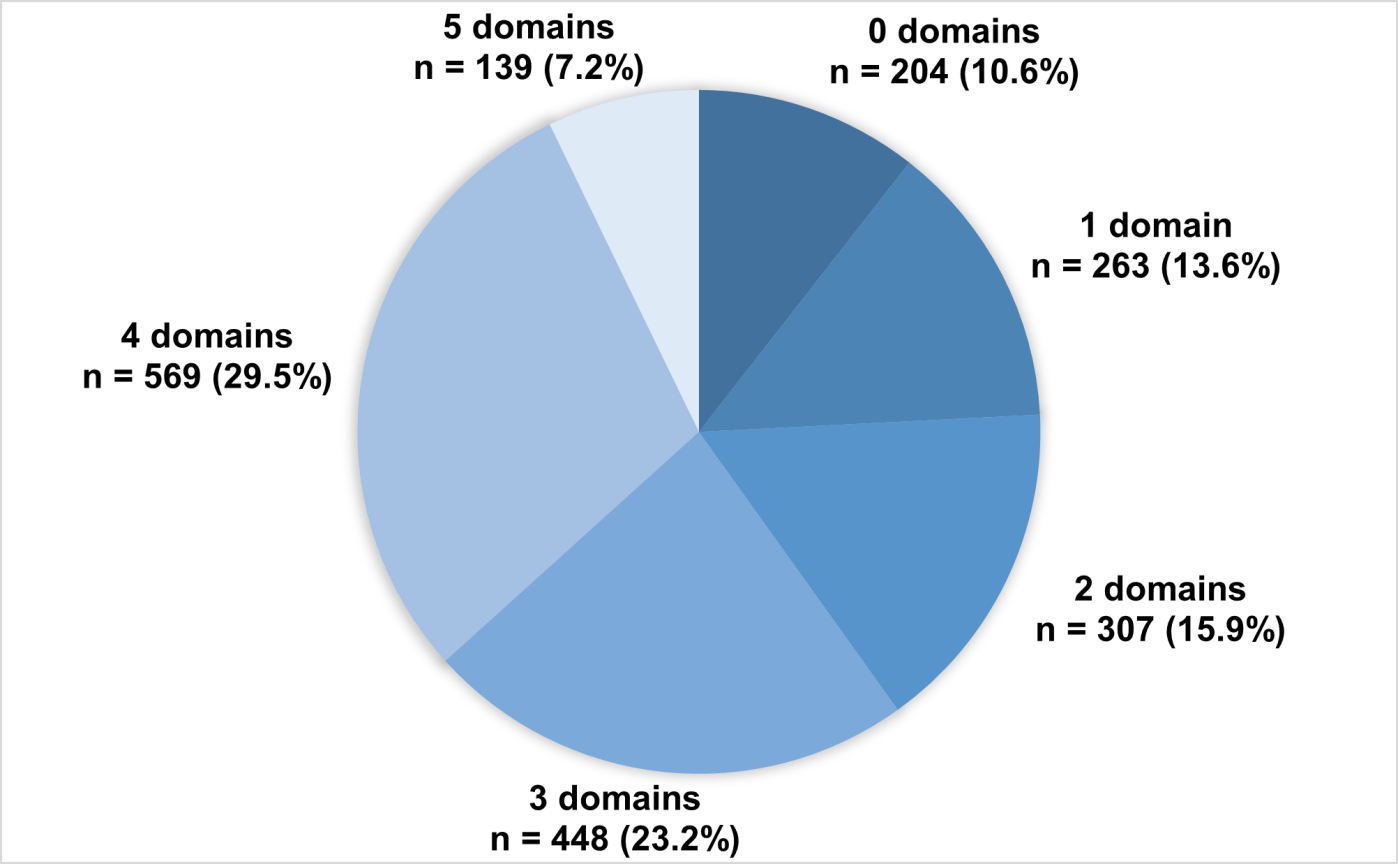
Cumulative breakdown of participant numbers meeting ‘caseness’ criteria across domains assessed via Hubs’ mental health and functional screening tools (depression, anxiety, post-traumatic stress, problematic alcohol use and functional impairment).

In terms of overall severity, 60% of hub clients scored in the most severe range of scores on at least one mental health screening measure (see table 1 for categories of severity and definition of caseness for each measure, eg, severe depression or anxiety; moderately severe or worse functional impairment; or possible alcohol dependence and online supplemental Table 1 for summarised data on overall severity aross measures). Only 10% of users presented scores in the lowest range of severity across all measures (eg, no depression; minimal anxiety; subthreshold for PTSD). As illustrated in figure 1, most participants had scores suggestive of multiple comorbid difficulties, with 60% of the sample meeting caseness criteria on at least three different screening measures.

## Factors associated with elevated mental health and functional difficulties

The results of the logistic regression analyses exploring factors associated with elevated mental health and functional difficulties among hub clients are summarised below and reported in full in online supplemental tables 2–7.

The regression analyses to identify factors associated with higher likelihood of PHQ-9 caseness found that having a disability (OR=1.71; 95% CI (1.19, 2.53), p=0.005), a minority sexual orientation (ie, participants identifying as any sexual orientation other than heterosexual; OR=1.89, 95% CI (1.23, 2.94), p=0.004), suffering a financial loss (OR=1.48; 95% CI (1.14, 1.95), p=0.004) and having prepandemic emotional well-being concerns (OR=2.03; 95% CI (1.62, 2.53), p<0.001) were associated with higher likelihood for caseness. Undertaking new work-related tasks was also associated with greater likelihood of caseness (OR=1.23; 95% CI (1.01, 1.51), p=0.038), with interaction analyses indicating more pronounced PHQ-9 caseness risk at site D relatively to other hubs (p<0.001).

The GAD-7 analyses found evidence of decreased likelihood of caseness with older age (OR=0.98; 95% CI (0.97, 0.99), p<0.001). Suffering a financial loss (OR=1.28; 95% CI (1.00, 1.64), p=0.049), having had a bereavement (OR=1.38; 95% CI (1.07, 1.77), p=0.012) and reporting prepandemic emotional well-being concerns (OR=2.05; 95% CI (1.66, 2.53), p<0.001) were associated with higher likelihood for caseness.

In terms of PTSD, working in ICU/critical care and having a disability was associated with higher likelihood of having PCL-5 scores suggestive of probable diagnosis for PTSD (OR=2.23; 95% CI (1.45, 3.52), p<0.001). Undertaking new tasks (OR=1.71; 95% CI (1.31, 2.25), p<0.001), moving to a new work location (OR=1.49; 95% CI (1.13, 1.95), p=0.004) and suffering a bereavement (OR=1.91; 95% CI (1.41, 2.58), p<0.001) were associated with higher likelihood of PTSD caseness on the ITQ. In both the PCL-5 and ITQ analyses, prepandemic emotional well-being concerns (OR=1.95; 95% CI (1.42, 2.70), p<0.001 and OR=1.59; 95% CI (1.20, 2.11), p=0.001, respectively) and suffering a financial loss (OR=1.72; 95% CI (1.12, 2.69), p=0.015 and OR=1.57; 95% CI (1.16, 2.13), p=0.003, respectively) were associated with increased likelihood of probable PTSD.

The AUDIT caseness analyses indicated that identifying as a man (OR=2.35; 95% CI (1.74, 3.16), p<0.001) and undertaking new tasks (OR=1.38; 95% CI (1.09, 1.76), p=0.008) were associated with increased risk for problematic alcohol use. Conversely, identifying as an ethnic minority (OR=0.24; 95% CI (0.09, 0.51), p=0.001), having a disability (OR=0.65; 95% CI (0.41, 0.98), p=0.049), having experienced a hospitalisation because of COVID (OR=0.20; 95% CI (0.05, 0.54), p=0.006) and moving to a new work location (OR=0.71; 95% CI (0.55, 0.93), p=0.001) were associated with lower risk for problematic alcohol use.

The analyses to identify factors associated with significant impairments in functioning found that identifying as any sexual orientation other than heterosexual (OR=2.44; 95% CI (1.45, 4.35), p=0.002), having a disability (OR=1.93; 95% CI (1.23, 3.15), p=0.006), having a family member recovering from COVID at home (OR=1.62; 95% CI (1.24, 2.14), p=0.001), suffering a financial loss (OR=1.59; 95% CI (1.17, 2.19), p=0.004) and prepandemic emotional well-being concerns (OR=2.29; 95% CI (1.77, 2.97), p<0.001) were associated with a higher likelihood of presenting with WSAS scores indicative of significant impairment in functioning.

The results of the proportional odds ordinal logistic regression analyses to identify factors associated with greater overall severity across the various mental health screening measures used by the hubs are displayed in online supplemental tables 8 and 9. In these analyses, ORs relate to the odds of being in a higher severity category (moderate, high) in the presence of the putative risk factor (or, for age, for each 1-year increase).

Age was negatively associated with severity rating, such that people with higher age tended to have lower overall severity ratings (OR=0.99; 95% CI (0.98, 1.00), p=0.05). Identifying as any sexual orientation other than heterosexual was associated with higher rating (OR=1.75; 95% CI (1.22, 2.63), p=0.004). The presence of a disability (OR=1.70; 95% CI (1.21, 2.41), p=0.003), a family member having COVID-19 and recovering at home (OR=1.31; 95% CI (1.06, 1.63), p=0.01), suffering financial loss (OR=1.84; 95% CI (1.43, 2.39), p<0.001) and prepandemic emotional well-being concerns (OR=2.11; 95% CI (1.72, 2.59), p<0.001) were associated with higher ratings. We did not find evidence that associations varied across hubs.

## Discussion

This study represents the first multisite evaluation of the demographic and occupational characteristics of clients who accessed Resilience Hub services dedicated to supporting the mental health needs of health and social care workers during the COVID-19 pandemic. The severity of, and factors associated with, common mental health difficulties among these help-seeking, high-risk occupational groups were explored to inform ongoing and future strategies for supporting the health and social care workforce.

The findings indicated that most hub clients who completed the hub screening offer worked in NHS healthcare settings, with considerably smaller proportions of respondents working for other in-scope sectors. Hub clients included in these analyses predominantly identified as women and from a white background. These figures are in contrast with workforce demographics across health and social care sector, whereby men typically make up 18% and 24% of the workforce for social care and the NHS, respectively.^21 22^ People identifying as from a black, Asian or minority ethnic background typically make up 23% and 30% of the workforce for social care and the NHS, respectively.^21 22^ It is unlikely that the observed difference between the demographics of our sample and those of the broader NHS and social care workforce could be entirely attributable to self-selection for the present analyses (ie, as participants consented for their anonymised data to be used for research purposes) or geographical variances. The findings are, therefore, suggestive that hub clients may under-represent specific demographic and occupational groups, including individuals from black, Asian and minority ethnic groups, men and people working in social care and emergency services. While some of these differences may be due to restrictions of support to certain groups as per evolving national guidance during the study, for example, around the inclusion of emergency service workers, as well as phased opening of offers that prioritised certain occupational groups*,* these findings highlight possible issues with the visibility and/or accessibility of hub support for certain in-scope occupational and demographic groups, which could be addressed as part of future initiatives to better target these under-represented groups. Qualitative findings from the wider mixed-methods study expand on potential barriers that different demographic and occupational groups experienced in accessing support during the pandemic.^14^ Barriers for staff from minoritised ethnic communities, for example, included being discouraged from accessing the hubs due to past negative experiences from other NHS services; limited representation of diversity on hub clinical teams; and a perception that hubs were less well equipped to support staff with the impact of racism.^14^ Barriers for other staff included limited accommodation for out-of-hours sessions for those doing shift work and lack of cover at work for care home staff.^14^

Participants presented with considerable mental health needs across all domains assessed. The prevalence of mental health difficulties was broadly comparable across hubs, but with slightly lower observed figures for site D but also marked differences in PTSD caseness between hubs that used different instruments to assess post-traumatic stress, that is, ITQ was associated with lower detected caseness relatively to PCL-5. Approximately 80% of hub clients had scores suggestive of significant impairments in functioning. Furthermore, 60% of hub clients scored in the most severe range of scores on at least one of the screening measures, while only 10% had subclinical scores across all measures. These figures are generally congruent with the findings of other research highlighting elevated mental health needs among health and social care staff during the COVID-19 pandemic as well as elevated prepandemic mental health risk in certain occupational groups (eg, healthcare workers).^23^ Nonetheless, the observed prevalence of significant difficulties in this study is striking, and likely due to the help-seeking nature of this sample. These findings, alongside data indicating that a considerable proportion of Hub clients reported being concerned about their emotional well-being prior to the pandemic, suggest that the hub clients presented with a degree of complexity, characterised by multiple co-occurring mental health difficulties which impacted functioning, as well as difficulties that may be long-lasting, that is, they may have preceded (and potentially aggravated by) the COVID-19 pandemic. Whilst our analyses did not account for temporal trends, it is possible that levels of ‘caseness’ may have varied, and potentially increased, over the course of the pandemic. This would be consistent with the relatively lower prevalence of difficulties observed at that became fully operational in earlier phases of the pandemic (eg, site D).

Our analyses identified several characteristics associated with clinically significant mental health concerns in this sample. Older age was found to be associated with reduced risk for anxiety and overall severity of presentations. Participants who described their ethnic background as white were at higher risk for problematic alcohol use. Individuals who identified as men had an elevated risk for alcohol-related problems. Hub clients who identified with any sexual orientation other than heterosexual were at elevated risk for depression, alcohol misuse, functional impairment and higher overall severity. Having a disability was associated with increased risk for depression, post-traumatic stress, functional impairment and higher overall severity but also a reduced risk for alcohol-related problems compared with participants who did not report any disability on the screening questionnaires. These findings are consistent with those of prior studies focusing on the association between these individual characteristics and mental health difficulties in both specific staff groups eligible for hub support (eg, healthcare workers) and the general population.^724^,^26^

While fine-grained analyses considering the relative risk of specific occupational characteristics were unviable (due to the heterogeneity in which this information was collected across sites), our analyses focusing on ICU/critical care workers (a particular ‘high-risk’ group due to their high level of disease exposure during the pandemic) found evidence suggestive of particularly elevated risk for post-traumatic stress. This finding is consistent with recent UK research reporting high levels of probable PTSD and other mental health difficulties in this group.^7^ Other occupational variables potentially associated with higher risk included specific stressful circumstances experienced during the pandemic. Being seconded or redeployed into different work roles was not associated with increased risk; this finding is surprising in that other pandemic literature demonstrates the negative impact of redeployment.^27^ However, the finding may be explained by the broad category of redeployment, as certain experiences of redeployment have been found to have a particularly negative mental health impact compared with others, including redeployment to ICU wards, or redeployment without adequate training.^28^ Moving to a new work location (a closely related variable) was associated with increased risk for PTSD, whereas undertaking new tasks was associated with increased risk for depression, post-traumatic stress and problematic alcohol use.

In line with findings from other research, other stressful life circumstances experienced during the pandemic also had an impact on the mental health difficulties reported by the present sample.^4 29^ Suffering a financial loss during the pandemic was (together with having prepandemic emotional well-being concerns) the most consistent variable associated with higher likelihood for caseness across all the domains assessed by the hub screening measures. Having recovered from severe COVID illness which involved hospitalisation and/or having a family member undergoing a similar adverse experience was associated with increased risk for post-traumatic stress. Conversely, having family members who recovered at home from COVID was associated with higher anxiety risk as well as greater functional impairment. Suffering a bereavement was associated with increased risk for anxiety and post-traumatic stress.

### Limitations

The study has some limitations, several due to the nature of using routinely collected data from clinical services. The implications of our research are limited by the lack of a comparison group, for example, exploring uptake of other support services in a region without hub support available. Likewise, while a high proportion of Hub clients gave consent for the use of their mental health screening data for research purposes, lack of consent precluded our ability to analyse the data to identify whether there were any differences between those who consented and those who did not. The findings report on mental health symptoms measured by standardised screening questionnaires, and while they are not taken in this study to represent psychiatric diagnoses, research suggests that such questionnaires may nevertheless overestimate the prevalence of mental health difficulties among healthcare staff during the pandemic.^30^ Our findings also suggest that the use of different instruments may substantially alter the observed prevalence of mental health difficulties in samples of health and social care workers. More specifically, while the ITQ and the PCL-5 are instruments designed to detect probable PTSD according to different diagnostic classification systems (International Classification of Diseases, ICD-11, and Diagnostic and Statistical Manual of Mental Disorders, Fifth Edition, DSM-5, respectively), it is likely that their observed incongruence in our data may stem from other factors. While some reports suggest good convergent validity between these PTSD screeners, other reports have considerable diagnostic disagreement between these two tools in certain samples,^31^ highlighting the need for further psychometric evaluation among health and social care workers. Finally, the current study explores 10% of the 40 hubs set up during the pandemic, and the NHS England guidance around the hubs’ setup was broad and has been operationalised with high levels of local variation across hubs, therefore, these findings may not be representative of all staff well-being hubs.

### Clinical implications

These findings further contextualise qualitative data from the wider mixed-methods evaluation of the hubs, which demonstrated that the hubs were particularly valued by staff as a support service that was separate from occupational health services and from their organisations’ patient records systems.^14^ The hubs offered systems of support that seem to have provided an important offer for health and social care staff with significant mental health needs who may have otherwise struggled to directly access other sources of support via primary or secondary mental healthcare services. The present data, alongside our previously published qualitative work,^14^ suggest an important need for services supporting these staff groups, in particular within the context of the multiple barriers to seeking and accessing mental health support that may be experienced by this population.^32^

While our analyses suggest important considerations in relation to how hub support might have reached certain occupational and ethnic minority groups less effectively, meaningful outreach and engagement with under-represented groups may help to address potential barriers to Hub service access in future.

While the acute impacts of the pandemic may no longer be perceived as urgently pressing on the well-being of health and social care staff, there is a clear and continued need to provide effective mental health and well-being support for health and social care staff. Although exacerbated by the pandemic, sickness absence due to mental health was already a pressing need prior to COVID-19,^33^ and currently the most common reason for sickness absence in the NHS (25% of all absences) is ‘anxiety/stress/depression/other psychiatric illness’.^33^ These challenges are likely to continue to increase, in light of extreme pressures on the workforce, including staff retention issues and increasingly high job vacancies, and the above evidence around the delays in staff’s help-seeking. On top of workforce issues, the cost-of-living crisis is also taking its toll on staff. Staff mental health and well-being support is therefore likely to continue to represent an important national challenge in the years to come, with potential indirect repercussions on the ability to deliver effective social and healthcare for the general population. Services like the Hubs could, pending further evaluation, represent an effective component of a broader response to this problem; however, this response relies on continued funding which is currently under threat now that national funding for hub services has ceased.

### Research implications

While the present work highlights the high levels of mental health needs among hub clients on registration with these services, future research should seek to establish the effectiveness of hub services, for example, through the longitudinal collection of mental health data for health and social care staff accessing hub support, and the systematic comparison of data from staff well-being and occupational outcomes (eg, severity of mental health difficulties; mental health work absences) in regions where hub support is available and regions that have no available hub support. As the availability of hub support may decrease due to loss of national funding to support them postpandemic, a large-scale naturalistic evaluation using a quasi-experimental design could be used to determine the clinical and cost-effectiveness of the model.

## Supplementary material

10.1136/bmjopen-2023-082817 online supplemental file 1

## Data Availability

Data are available on reasonable request.
