# Supplementary material for: Demographic, occupational factors and pandemic-related stressors associated with heightened mental health difficulties among UK health and social care workers supported by regional Resilience Hubs during the COVID-19 pandemic
Source: BMJ Open. 2025 Feb 25;15(2):e082817. doi: 10.1136/bmjopen-2023-082817 (PMC12083307; doi:10.1136/bmjopen-2023-082817)
Supplement: online supplemental file 1 [file bmjopen-15-2-s001.docx]

Supplementary material

**1. Definition of the “overall severity” variable**

The measure of ‘overall severity’ used in our regression analyses was defined from the screening questionnaires as the highest severity grade received across the questionnaires using the categorisation system illustrated in the Table below. If the scores across all available measure were consistent with the mildest severity classification for each measure, a person was categorised as presenting a “low” severity profile. If the person’s highest severity grade was consistent with those listed in the moderate column, a ‘moderate’ severity category was applied. If the person scored in the higher tiers of severity in at least one measure, a “high” severity classification was applied. Missing data was allowed on any measure, with overall severity being calculated from the available measures. Overall severity was set as missing if all measures had missing data for that individual.

Supplementary Table 1: Overall severity table

| **Overall severity** | **PHQ9** | **GAD7** | **PCL-5** | **ITQ** | **AUDIT** | **WSAS** |
| --- | --- | --- | --- | --- | --- | --- |
| **LOW** | None; Mind | None; Mild | No PTSD | No PTSD/CPTSD | Low risk | Subclinical |
| **MODERATE** | Moderate; Moderately severe | Moderate | n/a | n/a | Hazardous | Significant |
| **HIGH** | Severe | Severe | PTSD present | PTSD / CPTSD present | Harmful; Possible dependence | Moderately  severe or worse |

**2. Occupational characteristics of the sample**

Supplementary Table 2: N (%) for the occupational data of the sample.

|  | **Site A (n=475)** | **Site B  (n=367)** | **Site C (n=400)** | **Site D (n=731)** | **Total**  **(n=1973)** |
| --- | --- | --- | --- | --- | --- |
|  |  |  |  |  |  |
| NHS | 289 (60.2) | 315 (87.0) | 222 (57.8) | 312 (44.0) | 1138 (58.9) |
| Primary care | 31 (6.5) | 15 (4.1) | 20 (5.2) | 66 (9.3) | 132 (6.8) |
| Social care | 18 (3.8) | 13 (3.6) | 26 (6.5) | 59 (8.3) | 116 (6.0) |
| Emergency services | 20 (4.2) | 3 (0.8) | 45 (11.7) | 30 (4.2) | 98 (5.0) |
| Education | 14 (2.9) | 0 (0) | 1 (0.2) | 9 (1.3) | 24 (1.2) |
| VCSE | 2 (0.4) | 0 (0) | 13 (3.4) | 36 (5.1) | 51 (2.6) |
| Local authority | 17 (3.5) | 0 (0) | 4 (1) | 15 (2.1) | 36 (1.9) |
| Other^*^ | 84 (17.5) | 16 (4.4) | 54 (14.1) | 182 (25.7) | 336 (17.4) |
| Missing | *0% missing* | *1.4% missing* | *4% missing* | *3% missing* | *2.1% missing* |
|  |  |  |  |  |  |

*_Note:_* _All percentages calculated excluding missing values_

*In all sites other than Site D, free text information about job role were available, therefore it was often possible to re-categorise clients from ‘Other’ to one of the main reported categories included in the table, most commonly to the NHS category. However, this open text response option was not available for Site D, hence a high proportion of ‘Other’ job roles.

**3. Regression tables for the caseness and overall severity analyses**

Supplementary Table 3: Summary of logistic regression analyses of PHQ-9 caseness

| **Predictor** | **OR** | **95% CI** | | **p** | **Interaction p-value** |
| --- | --- | --- | --- | --- | --- |
| *Demographics* |  |  |  |  |  |
| Age | 1 | 0.99 | 1.01 | 0.416 | 0.525 |
| Gender (man vs woman) | 1.02 | 0.77 | 1.36 | 0.879 | 0.744 |
| Gender (identified in another way vs woman) | 0.75 | 0.36 | 1.62 | 0.507 | - |
| Ethnicity (ethnic minority vs white) | 0.66 | 0.43 | 1.03 | 0.063 | 0.044 |
| ICU/critical care | 1.14 | 0.81 | 1.64 | 0.458 | 0.466 |
| Clinical vs non-clinical | 0.67 | 0.44 | 1.01 | 0.062 | 0.024 |
| Sexual orientation (identified in another way vs heterosexual) | 1.89 | 01.23 | 2.94 | 0.004 | 0.969 |
| Disability | 1.71 | 1.19 | 2.53 | 0.005 | 0.264 |
| *Impacts of COVID* |  |  |  |  |  |
| COVID illness (home) | 1.21 | 0.97 | 1.5 | 0.094 | 0.266 |
| COVID illness (hospital) | 1.32 | 0.74 | 2.48 | 0.364 | 0.483 |
| COVID family member (home) | 1.21 | 0.96 | 1.52 | 0.11 | 0.228 |
| COVID family member (hospital) | 1.06 | 0.74 | 1.54 | 0.763 | 0.995 |
| Financial loss | 1.48 | 1.14 | 1.95 | 0.004 | 0.489 |
| Undertaking new tasks | 1.23 | 1.01 | 1.51 | 0.038 | <0.001 |
| Seconded or re-deployed | 0.93 | 0.71 | 1.23 | 0.616 | 0.661 |
| Moved work location | 1.22 | 0.98 | 1.53 | 0.076 | 0.211 |
| Bereavement | 1.26 | 0.97 | 1.64 | 0.089 | 0.242 |
| *Pre-pandemic MH concerns* |  |  |  |  |  |
| Yes (vs no) | 2.03 | 1.62 | 2.53 | <0.001 | 0.085 |
| Unsure (vs no) | 1.81 | 1.37 | 2.42 | 0.001 | - |

Supplementary Table 4: Summary of logistic regression analyses of GAD-7 caseness

| **Predictor** | **OR** | **95% CI** | | **p** | **Interaction p-value** |
| --- | --- | --- | --- | --- | --- |
| *Demographics* |  |  |  |  |  |
| Age | 0.98 | 0.97 | 0.99 | <0.001 | 0.576 |
| Gender (man vs woman) | 0.95 | 0.73 | 1.25 | 0.725 | 0.726 |
| Gender (identified in another way vs woman) | 0.78 | 0.37 | 1.64 | 0.507 | - |
| Ethnicity (ethnic minority vs white) | 1.14 | 0.74 | 1.79 | 0.547 | 0.432 |
| ICU/critical care | 1.15 | 0.83 | 1.6 | 0.406 | 0.843 |
| Clinical vs non-clinical | 0.98 | 0.67 | 1.41 | 0.899 | 0.689 |
| Sexual orientation (identified in another way vs heterosexual) | 1.32 | 0.92 | 1.92 | 0.13 | 0.477 |
| Disability | 1.17 | 0.85 | 1.63 | 0.33 | 0.230 |
| *Impacts of COVID* |  |  |  |  |  |
| COVID illness (home) | 0.9 | 0.74 | 1.1 | 0.311 | 0.003 |
| COVID illness (hospital) | 0.82 | 0.49 | 1.38 | 0.445 | 0.68 |
| COVID family member (home) | 1.13 | 0.91 | 1.4 | 0.272 | 0.001 |
| COVID family member (hospital) | 1.39 | 0.97 | 2.01 | 0.074 | 0.715 |
| Financial loss | 1.28 | 1 | 1.64 | 0.049 | 0.649 |
| Undertaking new tasks | 1.13 | 0.94 | 1.37 | 0.194 | 0.583 |
| Seconded or re-deployed | 0.92 | 0.71 | 1.19 | 0.521 | 0.494 |
| Moved work location | 1.21 | 0.98 | 1.49 | 0.074 | 0.192 |
| Bereavement | 1.38 | 1.07 | 1.77 | 0.012 | 0.613 |
| *Pre-pandemic MH concerns* |  |  |  |  |  |
| Yes (vs no) | 2.05 | 1.66 | 2.53 | <0.001 | 0.399 |
| Unsure (vs no) | 1.66 | 1.28 | 2.17 | 0.001 | - |

Supplementary Table 5: Summary of logistic regression analyses of PTSD caseness based on the PCL-5

| **Predictor** | **OR** | **95% CI** | | **p** | **Interaction p-value** |
| --- | --- | --- | --- | --- | --- |
| *Demographics* |  |  |  |  |  |
| Age | 1 | 0.99 | 1.01 | 0.927 | 0.73 |
| Gender (man vs woman) | 1.09 | 0.73 | 1.65 | 0.681 | 0.633 |
| Gender (identified in another way vs woman) * | - | - | - | - | - |
| Ethnicity (ethnic minority vs white) | 1.89 | 0.93 | 4.15 | 0.093 | 0.296 |
| ICU/critical care | 2.23 | 1.45 | 3.52 | <0.001 | 0.536 |
| Clinical vs non-clinical | 0.92 | 0.49 | 1.67 | 0.781 | NA** |
| Sexual orientation (identified in another way vs heterosexual) | 1.59 | 0.99 | 2.63 | 0.062 | 0.627 |
| Disability | 1.79 | 1.12 | 2.94 | 0.018 | 0.384 |
| *Impacts of COVID* |  |  |  |  |  |
| COVID illness (home) | 1.00 | 0.73 | 1.37 | 0.998 | 0.821 |
| COVID illness (hospital) | 2.56 | 1.09 | 7.02 | 0.044 | 0.436 |
| COVID family member (home) | 0.98 | 0.7 | 1.37 | 0.898 | 0.273 |
| COVID family member (hospital) | 1.27 | 0.7 | 2.38 | 0.445 | 0.227 |
| Financial loss | 1.72 | 1.12 | 2.69 | 0.015 | 0.019 |
| Undertaking new tasks | 0.97 | 0.73 | 1.29 | 0.826 | 0.004 |
| Seconded or re-deployed | 1.05 | 0.73 | 1.52 | 0.797 | 0.390 |
| Moved work location | 0.98 | 0.71 | 1.37 | 0.928 | 0.762 |
| Bereavement | 1.48 | 0.97 | 2.29 | 0.072 | 0.030 |
| *Pre-pandemic MH concerns* |  |  |  |  |  |
| Yes (vs no) | 1.95 | 1.42 | 2.7 | <0.001 | 0.623 |
| Unsure (vs no) | 1.29 | 0.87 | 1.91 | 0.205 | - |

***^Note^***^: * This model was not computable due to small numbers ** No interaction was computable as PCL-5 data were available for one site only^

Supplementary Table 6: Summary of logistic regression analyses of PTSD caseness based on the ITQ

| **Predictor** | **OR** | **95% CI** | | **p** | **Interaction p-value** |
| --- | --- | --- | --- | --- | --- |
| *Demographics* |  |  |  |  |  |
| Age | 0.99 | 0.98 | 1 | 0.191 | 0.923 |
| Gender (man vs woman) | 1.17 | 0.82 | 1.67 | 0.381 | 0.818 |
| Gender (identified in another way vs woman) | 1.13 | 0.46 | 2.55 | 0.783 | - |
| Ethnicity (ethnic minority vs white) | 1.32 | 0.74 | 2.3 | 0.333 | 0.263 |
| ICU/critical care | 1.44 | 0.83 | 2.45 | 0.184 | 0.844 |
| Clinical vs non-clinical | 1.1 | 0.69 | 1.8 | 0.686 | 0.239 |
| Sexual orientation (identified in another way vs heterosexual) | 1.20 | 0.70 | 2.00 | 0.501 | 0.211 |
| Disability | 1.32 | 0.84 | 2.03 | 0.22 | 0.522 |
| *Impacts of COVID* |  |  |  |  |  |
| COVID illness (home) | 0.83 | 0.62 | 1.09 | 0.187 | 0.504 |
| COVID illness (hospital) | 1.25 | 0.6 | 2.49 | 0.539 | 0.121 |
| COVID family member (home) | 1.27 | 0.96 | 1.67 | 0.097 | 0.639 |
| COVID family member (hospital) | 1.62 | 1.06 | 2.48 | 0.025 | 0.596 |
| Financial loss | 1.57 | 1.16 | 2.13 | 0.003 | 0.382 |
| Undertaking new tasks | 1.71 | 1.31 | 2.25 | <0.001 | 0.713 |
| Seconded or re-deployed | 1.39 | 0.97 | 1.99 | 0.07 | 0.406 |
| Moved work location | 1.49 | 1.13 | 1.95 | 0.004 | 0.043 |
| Bereavement | 1.91 | 1.41 | 2.58 | <0.001 | 0.314 |
| *Pre-pandemic MH concerns* |  |  |  |  |  |
| Yes (vs no) | 1.59 | 1.20 | 2.11 | 0.001 | 0.34 |
| Unsure (vs no) | 1.07 | 0.73 | 1.55 | 0.72 | - |

Supplementary Table 7: - Summary of logistic regression analyses of AUDIT caseness

| **Predictor** | **OR** | **95% CI** | | **p** | **Interaction p-value** |
| --- | --- | --- | --- | --- | --- |
| *Demographics* |  |  |  |  |  |
| Age | 1 | 0.99 | 1.01 | 0.909 | 0.553 |
| Gender (man vs woman) | 2.35 | 1.74 | 3.16 | <0.001 | 0.291 |
| Gender (identified in another way vs woman) | 1.40 | 0.54 | 3.21 | 0.455 | - |
| Ethnicity (ethnic minority vs white) | 0.24 | 0.09 | 0.51 | 0.001 | 0.151 |
| ICU/critical care | 1.43 | 0.98 | 2.08 | 0.061 | 0.009 |
| Clinical vs non-clinical | 1.35 | 0.87 | 2.16 | 0.19 | 0.004 |
| Sexual orientation (identified in another way vs heterosexual) | 1.47 | 0.95 | 2.22 | 0.072 | 0.167 |
| Disability | 0.65 | 0.41 | 0.98 | 0.049 | 0.214 |
| *Impacts of COVID* |  |  |  |  |  |
| COVID illness (home) | 1.07 | 0.83 | 1.37 | 0.622 | 0.77 |
| COVID illness (hospital) | 0.2 | 0.05 | 0.54 | 0.006 | 0.329 |
| COVID family member (home) | 1.1 | 0.84 | 1.42 | 0.488 | 0.476 |
| COVID family member (hospital) | 0.74 | 0.46 | 1.15 | 0.2 | 0.568 |
| Financial loss | 1.17 | 0.87 | 1.55 | 0.291 | 0.807 |
| Undertaking new tasks | 1.38 | 1.09 | 1.76 | 0.008 | 0.627 |
| Seconded or re-deployed | 0.93 | 0.67 | 1.27 | 0.648 | 0.651 |
| Moved work location | 0.71 | 0.55 | 0.93 | 0.012 | 0.943 |
| Bereavement | 1.3 | 0.97 | 1.73 | 0.07 | 0.136 |
| *Pre-pandemic MH concerns* |  |  |  |  |  |
| Yes (vs no) | 1.18 | 0.90 | 1.53 | 0.226 | 0.018 |
| Unsure (vs no) | 1.53 | 1.12 | 2.09 | 0.008 | - |

Supplementary Table 8: Part 2- Summary of logistic regression analyses of WSAS caseness

| **Predictor** | **OR** | **95% CI** | | **p** | **Interaction p-value** |
| --- | --- | --- | --- | --- | --- |
| *Demographics* |  |  |  |  |  |
| Age | 0.99 | 0.98 | 1 | 0.198 | 0.061 |
| Gender (man vs woman) | 1.12 | 0.81 | 1.56 | 0.498 | 0.498 |
| Gender (identified in another way vs woman) | 1.42 | 0.6 | 3.88 | 0.456 | - |
| Ethnicity (ethnic minority vs white) | 0.87 | 0.54 | 1.45 | 0.568 | 0.481 |
| ICU/critical care | 0.85 | 0.59 | 1.26 | 0.409 | 0.674 |
| Clinical vs non-clinical | 0.66 | 0.41 | 1.03 | 0.078 | 0.2 |
| Sexual orientation (identified in another way vs heterosexual) | 2.44 | 1.45 | 4.35 | 0.002 | 0.189 |
| Disability | 1.93 | 1.23 | 3.15 | 0.006 | 0.190 |
| *Impacts of COVID* |  |  |  |  |  |
| COVID illness (home) | 1.23 | 0.96 | 1.59 | 0.1 | 0.576 |
| COVID illness (hospital) | 1.26 | 0.66 | 2.67 | 0.513 | 0.882 |
| COVID family member (home) | 1.62 | 1.24 | 2.14 | 0.001 | 0.473 |
| COVID family member (hospital) | 1.06 | 0.71 | 1.64 | 0.772 | 0.628 |
| Financial loss | 1.59 | 1.17 | 2.19 | 0.004 | 0.912 |
| Undertaking new tasks | 1.13 | 0.9 | 1.41 | 0.295 | 0.129 |
| Seconded or re-deployed | 0.83 | 0.62 | 1.13 | 0.237 | 0.195 |
| Moved work location | 1.06 | 0.83 | 1.36 | 0.643 | 0.839 |
| Bereavement | 1.08 | 0.81 | 1.45 | 0.595 | 0.173 |
| *Pre-pandemic MH concerns* |  |  |  |  |  |
| Yes (vs no) | 2.29 | 1.77 | 2.97 | <0.001 | 0.018 |
| Unsure (vs no) | 1.71 | 1.25 | 2.37 | 0.001 | - |

Supplementary Table 9: Part 2 - Summary of proportional odds logistic regression analyses of overall severity across the Hub screening measures

| **Predictor** | **OR** | **95% CI** | | **p** | **Interaction p-value** |
| --- | --- | --- | --- | --- | --- |
| *Demographics* |  |  |  |  |  |
| Age | 0.99 | 0.98 | 1.00 | 0.05 | 0.91 |
| Gender (man vs woman) | 1.07 | 0.82 | 1.40 | 0.62 | 0.83 |
| Gender (identified in another way vs woman) | 1.07 | 0.52 | 2.25 | 0.86 | - |
| Ethnicity (ethnic minority vs white) | 0.85 | 0.56 | 1.32 | 0.47 | 0.19 |
| ICU/critical care | 1.28 | 0.92 | 1.81 | 0.15 | 0.60 |
| Clinical vs non-clinical* | 0.81 | 0.56 | 1.16 | 0.26 | Not computable |
| Sexual orientation (identified in another way vs heterosexual) | 1.75 | 1.22 | 2.63 | 0.004 | 0.28 |
| Disability | 1.70 | 1.21 | 2.41 | 0.003 | 0.58 |
| *Impacts of COVID* |  |  |  |  |  |
| COVID illness (home) | 1.18 | 0.97 | 1.45 | 0.11 | 0.19 |
| COVID illness (hospital) | 1.44 | 0.83 | 2.61 | 0.21 | 0.93 |
| COVID family member (home) | 1.31 | 1.06 | 1.63 | 0.01 | 0.13 |
| COVID family member (hospital) | 1.18 | 0.83 | 2.61 | 0.21 | 0.82 |
| Financial loss | 1.84 | 1.43 | 2.39 | <0.001 | 0.92 |
| Undertaking new tasks | 1.19 | 0.99 | 1.44 | 0.06 | 0.04 |
| Seconded or re-deployed | 1.04 | 0.81 | 1.35 | 0.76 | 0.42 |
| Moved work location | 1.15 | 0.94 | 1.41 | 0.19 | 0.22 |
| Bereavement | 1.25 | 0.98 | 1.60 | 0.07 | 0.30 |
| *Pre-pandemic MH concerns* |  |  |  |  |  |
| Yes vs no | 2.11 | 1.72 | 2.59 | <0.001 | 0.15 |
| Yes vs unsure | 1.43 | 1.08 | 1.90 | 0.01 | - |

* It was not possible to adjust this analysis for site due to the distribution of the outcome across sites in the subgroup of NHS workers. Attempting to do so resulted in non-convergence of the model.
