# Supplementary material for: Demographic, occupational factors and pandemic-related stressors associated with heightened mental health difficulties among UK health and social care workers supported by regional Resilience Hubs during the COVID-19 pandemic
Source: BMJ Open. 2025 Feb 25;15(2):e082817. doi: 10.1136/bmjopen-2023-082817 (PMC12083307; doi:10.1136/bmjopen-2023-082817)
Supplement: Supplementary file 2 [file bmjopen-2023-082817.reviewer_comments.pdf]

PEER REVIEW HISTORY

BMJ Open publishes all reviews undertaken for accepted manuscripts. Reviewers are asked to complete a checklist review form and are provided with free text boxes to elaborate on their assessment. These free text comments are reproduced below.

ARTICLE DETAILS

Title (Provisional)

Demographic, occupational factors and pandemic-related stressors associated with heightened mental health difficulties amongst UK health and social care workers supported by regional Resilience Hubs during the COVID-19 pandemic

Authors

Varese, Filippo; Allsopp, Kate; Carter, Lesley-Anne; Wilkinson, Jack; Shields, Gemma E; Rowlandson, Aleix; Chung, Priscilla; Hassan, Alysha A.; White, Hannah; Wright, Sally-Anne; Young, Ellie; Davey, Jess; Barrett, Alan; Bhutani, Gita; Hind, Daniel; McGuirk, Katherine; Huntley, Fay; Sarsam, May; Walker, Holly; Jordan, Joanne; Ten Cate, Hein; Watson, Ruth; Willbourn, Jenni; French, Paul

---

VERSION 1 - REVIEW

**Reviewer**	**1**
**Name**	**Carbonell, Marina**
**Affiliation**	**Memorial University of Newfoundland, School of Maritime Studies**
**Date**	**02-Feb-2024**
**COI**	**N/A**

Title: Demographic, occupational factors and pandemic-related stressors associated with heightened mental health difficulties amongst UK health and social care workers supported by regional Resilience Hubs during the COVID-19 pandemic
BMJ Open, Manuscript bmjopen-2023-082817

Review

General: This manuscript describes the demographic characteristics of health and social care workers in England utilizing Resilience Hubs during the COVID-19 pandemic and reported mental health difficulties. Detailed comments/suggestions are located below.

Introduction

"Their aim was to expedite access to evidence-based mental health support that might have been otherwise be more challenging to access by health and social care professionals due to known barriers to help-seeking and the additional strain placed on services by the pandemic.1" Please clarify this sentence (p. 5, Line 33-38).

What is NICE guidance?

Methods

"All individuals screened by the Hubs were routinely asked to provide consent for their anonymised data to be used for research purposes." (p9, line 5-6). Could the authors clarify if this was an ongoing request for consent as each screening tool was completed or at specific intervals? Were participants given the option to withdraw from the study by any specific time point, and did any participants choose to withdraw from the study? Were there any exclusion criteria? Further information regarding the consent process is needed.

Results

"Of note, these differences may be artefactual and due to variances in how questions on disability status were framed at different Hubs." How were they framed differently, or how is disability defined for the data collection?

Here, Supplementary Table 2 describes the Occupational Data of the sample by site; however, this is limited in the results. If authors are unable to explore any differences in presentation of caseness by occupation type, this should be noted here. It is addressed in the Discussion briefly; however, the specific barriers to analysis are worth noting in the results because it reads as a gap when the analysis is limited by the data collection. The title of the article implies more exploration of the occupational factors in the manuscript; if any analysis is possible, it may be helpful to include.

Page 14, Line 10-17 "The prevalence of mental health difficulties was broadly comparable across Hubs, but with slightly lower observed figures for Site D but also marked differences in PTSD caseness between Hubs that used different instruments to assess post-traumatic stress i.e., ITQ was associated with lower detected caseness relatively to PCL-5" This is an important discussion point but the sentence needs clarification and further exploration in the discussion.

Discussion

The authors discuss the associations to risk; however, this section may benefit from further contextualization and interpretation of findings throughout the discussion section. For example, *why* were redeployment into different work roles or being seconded not associated with increased risk versus moving to a new work location?

The difference between study participants and workforce demographics is interesting but may be further explored in terms of access, existing personal supports, and stigma. Was there a barrier to access in terms of locations or was virtual access possible? Why would these Hubs be predominately white women? Is there any information on the staff of the Hubs, and their demographics?

Are all the occupation types availing of Hub services employed by NHS? Are they employed by counties/regions/towns, and would that have an impact on services available to them or the differences in work that they do? Are they unionized or do they have employee assistance programs through work?

Was the ability to use the Hub services contingent on showing support need? For example, are users required to do the diagnostic assessments as part of intake to receive services from a Hub, or is it possible to receive Hub services without formal assessments? What were the training qualifications of the mental health assessment administrators?

Limitations

"The implications of our limited by the lack of a comparison group, for example, exploring uptake of other support services in a region without Hub support available." P. 16, Line 12-15, This sentence is missing a word, or clarification is needed.

p. 16, Line 15-22 "Likewise, whilst a high proportion of Hub clients gave consent for the use of their mental health screening data for research purposes, lack of consent precluded our ability to analyse the data to identify consentthere were any differences between those who consented and those who did not." This sentence needs clarification – it implies some participants did not give consent. Please clarify the consent process (as mentioned above), and how the data was collected.

I would caution the authors in switching back and forth between 'staff' and 'clients' because, in some areas, it reads as Hub staff (i.e. the people working in the Hub) vs. participants of the study/clients/respondents (i.e. the people availing of Hub services). I agree that Hub users are all health and social staff, but in some areas of the paper, it reads less clearly than intended.

Reviewer 2

Name Fisher, Jane

Affiliation Monash University, Jean Hailes Research Unit, School of Public Health and Preventative Medicine

Date 04-Aug-2024

COI I have no competing interests

It is agreed that the pandemic and the restrictions imposed to limit spread of COVID-19 had adverse consequences for population-wide psychological functioning. These were especially burdensome for people providing frontline health and emergency care services. Different mechanisms were implemented in different nations to assist. Although, with the advent of vaccines, restrictions are now mostly removed, it is of relevance to future pandemic preparedness to learn which models were effective and for which sub-populations.

This paper reports an analysis of routinely collected data about nature and severity of psychological symptoms among health and social care professionals accessing a mental health service (Hubs) established in some regions in England.

The paper reads as parochial, with presumptions that an international readership will know about the National Health Service, Mental Health and Wellbeing Hubs, the Manchester Arena bombing and what NICE is. If these descriptors are used, they each need to be defined or described in sufficient detail, including for those in less well-resourced countries to identify feasibility or value of establishing equivalent services.

Specific gaps include:

1. The Introduction needs to provide a clear argument for a knowledge gap and then provide a specific aim for how this sub-study will address it. Please re-work it and add a specific rationale and aim.

2. All the details about the study should be in the Methods and not in the Introduction. The Methods need to follow a clear account of what was done and not to refer to what was found. All details about the participants should be placed in the Results.

3. Please explain who in this service is categorised as a health or social care professional. Please explain whether administrative or ancillary staff were included or excluded from this service.

4. It is not clear what the mental health service comprised. Please provide details of staffing, funding, whether it was a physical facility, the number of sessions available to individuals, what the 'evidence-based' treatments were, whether there was access 24 hours a day to enable shift workers to attend or only office hours, and what NICE Guidance recommends for occupational mental health in this circumstance.

5. A description of the funding should be put in the appropriate place at the end of the manuscript, not in the middle of the Introduction.

6. The Procedure needs to be described separately from the measures (better titled data sources), please make these distinct.

7. It is surprising to read that clients were 'required' to complete any information when earlier it is described as a voluntary self-referral process. Please make it clear whether providing information was a hurdle requirement for receiving care?

8. The explanation of how missing data were managed needs to be in the Methods not at the beginning of the Results.

9. If the Hubs were an NHS service it would be expected that most clients were NHS employees – who were the other clients and how were they given access?

10. What is meant by the term 'caseness' – presumably everyone seeking care at the hubs was categorised as a 'case' for administrative purposes.

11. 'emotional concerns' is quite a non-specific descriptor and not helpful for service planners elsewhere seeking to identify people at higher need. Please provide a more specific account of what these were and how they were ascertained.

12. As this was a service for mental health support, it is not surprising that clients had scores outside the average ranges. However, no effort is made to compare with data generated either in the UK or elsewhere about equivalent sub-population or general population data which would assist.

13. No effort is made to estimate what fraction of the workforce accessed the hubs, but this is essential in advocating for equivalent services elsewhere. Please add this information.

14. … between 84% of clients identified as … the other alternative is missing

15. The analyses appeared to be to identify the characteristics of people with the most severe symptoms, but it was not clear what the purpose of doing this was? This information could carry risks for potential employees that those with a history of mental health problems might be considered less employable because deteriorations in mental health and functioning occurred in response to the extra demands of the pandemic. This potential harm of the study should be considered in the Discussion.

16. The sample is selected and unrepresentative on several grounds. It could only include people who went to Hubs, and among these, only those who consented. It is well known that service users differ from the general population, and that only limited generalisation can be made. These biases mean that few generalisations can be made to the wider workforce or to other settings and in my opinion the implications should be framed very cautiously and be carefully qualified.

18. No outcome or economic data which might indicate the impact or cost-effectiveness of these services or consumer-based evaluations are provided. In the absence of these it is difficult to establish the worth of the data to an international readership or to health policymakers and service planners in other settings. In the absence of any prospective or other evaluation data it seems premature and speculative to suggest that these services were beneficial to mental health. These limitations should be considered in the Discussion.

19. Sex and gender are not interchangeable terms. In this paper it appears that categorisation was done on the basis of sex (woman / man) and this should be corrected throughout.

VERSION 1 - AUTHOR RESPONSE

REVIEWER 1

General:

Introduction

> *"Their aim was to expedite access to evidence-based mental health support that might have been otherwise be more challenging to access by health and social care professionals due to known barriers to help-seeking and the additional strain placed on services by the pandemic.1" Please clarify this sentence (p. 5, Line 33-38).*

This sentence has now been clarified.

> *What is NICE guidance?*

NICE is part of the publicly funded nationalised healthcare system in the UK, the National Health Service (NHS). For clarity, and in response to both reviewers' queries about NICE, we have amended this to read 'NHS guidance'.

Methods

> *"All individuals screened by the Hubs were routinely asked to provide consent for their anonymised data to be used for research purposes." (p9, line 5-6). Could the authors clarify if this was an ongoing request for consent as each screening tool was completed or at specific intervals? Were participants given the option to withdraw from the study by any specific time point, and did any participants choose to withdraw from the study? Were there any exclusion criteria? Further information regarding the consent process is needed.*

Hub clients completed all the screening measures at one time point upon self-referral to Hubs, therefore consent was asked at that one time point only as part of the routinely collected data. However, at any time point up until the final data extraction for the study (31st December 2021) Hub clients could ask the Hub to withdraw their consent. It is not known how many withdrew their consent between self-referral and data lock, because this process was managed by Hub teams, and final datasets were only transferred to research team at the end of data lock, at which point only data for Hub clients who had granted their consent was included. No data was transferred outside of Hubs for clients who had not given their consent to share data for research purposes.

We have clarified this information in the Procedures section, and added a note regarding exclusion criteria in the Participants section. Furthermore, we have added a sentence at the start of the Results section to highlight how many Hub clients gave consent for their data use; this information was previously only in the Strengths & Limitations bullet points at the start of the article.

Results

> *"Of note, these differences may be artefactual and due to variances in how questions on disability status were framed at different Hubs." How were they framed differently, or how is disability defined for the data collection?*

At Sites B and D, items to confirm lack of a disability (i.e., 'none') were embedded within an extensive, alphabetically ordered list of potential disabilities, which may have led to high levels of missingness (80.4% and 67% respectively). It is therefore likely that a considerable proportion of missing responses at these sites are from people without a disability, and disability data were analysed accordingly in our analyses. This has been briefly clarified in the amended manuscript.

> *Here, Supplementary Table 2 describes the Occupational Data of the sample by site; however, this is limited in the results. If authors are unable to explore any differences in presentation of caseness by occupation type, this should be noted here. It is addressed in the Discussion briefly; however, the specific barriers to analysis are worth noting in the results because it reads as a gap when the analysis is limited by the data collection. The title of the article implies more exploration of the occupational factors in the manuscript; if any analysis is possible, it may be helpful to include.*

Given the substantial preponderance of NHS employees amongst Hub clients and the small representation of certain occupational sectors in the available data, our analyses aimed at identifying occupational variables associated with greater mental health needs focused on more specific occupational variables that may convey heightened risk (e.g. working in high-risk settings like ICUs/critical care) as opposed to broad occupational sectors. This has been clarified in the amended manuscript.

> *Page 14, Line 10-17 "The prevalence of mental health difficulties was broadly comparable across Hubs, but with slightly lower observed figures for Site D but also marked differences in PTSD caseness between Hubs that used different instruments to assess post-traumatic stress i.e., ITQ was associated with lower detected caseness relatively to PCL-5" This is an important discussion point but the sentence needs clarification and further exploration in the discussion.*

This is already clarified in the Discussion section of our original manuscript, which stated: "Our findings also suggest that the use of different instruments may substantially alter the observed prevalence of mental health difficulties in samples of health and social care workers. More specifically, while the ITQ and the PCL-5 are instruments designed to detect probable PTSD according to different diagnostic classification systems (ICD-11 and DSM-5, respectively), it is likely that their observed incongruence in our data may stem from other factors. While some reports suggest good convergent validity between these PTSD screeners, other reports have considerable diagnostic disagreement between these two tools in certain samples,[28] highlighting the need for further psychometric evaluation amongst health and social care workers."

Discussion

> *The authors discuss the associations to risk; however, this section may benefit from further contextualization and interpretation of findings throughout the discussion section. For example, why were redeployment into different work roles or being seconded not associated with increased risk versus moving to a new work location? The difference between study participants and workforce demographics is interesting but may be further explored in terms of access, existing personal supports, and stigma. Was there a barrier to access in terms of locations or was virtual access possible? Why would these Hubs be predominately white women? Is there any information on the staff of the Hubs, and their demographics? Are all the occupation types availing of Hub services employed by NHS? Are they employed by counties/regions/towns, and would that have an impact on services available to them or the differences in work that they do? Are they unionized or do they have employee assistance programs through work? Was the ability to use the Hub services contingent on showing support need? For example, are users required to do the diagnostic assessments as part of intake to receive services from a Hub, or is it possible to receive Hub services without formal assessments? What were the training qualifications of the mental health assessment administrators?*

We have now updated the Discussion section to provide links to the literature on redeployment, and have cited our previously published qualitative work (Allsopp et al., 2023) which provides further context to barriers to support access for different demographic and occupational

groups. We have included a few examples in the Discussion, including barriers for staff from minoritised ethnic communities, shift workers, and care home staff.

Limitations

> *“The implications of our limited by the lack of a comparison group, for example, exploring uptake of other support services in a region without Hub support available.” P. 16, Line 12-15, This sentence is missing a word, or clarification is needed.*

We have amended this sentence accordingly.

> *p. 16, Line 15-22 “Likewise, whilst a high proportion of Hub clients gave consent for the use of their mental health screening data for research purposes, lack of consent precluded our ability to analyse the data to identify consent there were any differences between those who consented and those who did not.” This sentence needs clarification – it implies some participants did not give consent. Please clarify the consent process (as mentioned above), and how the data was collected.*

We believe this section has now been clarified as a result of the previously discussed amendments to the explanation of consent procedures, and the information regarding proportion of Hub clients consenting to data use provided at the start of the Results section.

> *I would caution the authors in switching back and forth between ‘staff’ and ‘clients’ because, in some areas, it reads as Hub staff (i.e. the people working in the Hub) vs. participants of the study/clients/respondents (i.e. the people availing of Hub services). I agree that Hub users are all health and social staff, but in some areas of the paper, it reads less clearly than intended.*

We have now amended the paper to use ‘Hub clients’ or ‘participants’ throughout from the Methods section onwards, and have added a statement to this effect in the Participants section.

REVIEWER 2:

> *The paper reads as parochial, with presumptions that an international readership will know about the National Health Service, Mental Health and Wellbeing Hubs, the Manchester Arena bombing and what NICE is. If these descriptors are used, they each need to be defined or described in sufficient detail, including for those in less well-resourced countries to identify feasibility or value of establishing equivalent services.*

The above terms have now been described in the paper, which the exception of NICE which has been changed to NHS guidance for clarity.

> Specific gaps include:
>
> *1. The Introduction needs to provide a clear argument for a knowledge gap and then provide a specific aim for how this sub-study will address it. Please re-work it and add a specific rationale and aim.*

We have now updated the introduced and clarified the rationale, aims, and objectives for the paper.

2. All the details about the study should be in the Methods and not in the Introduction. The Methods need to follow a clear account of what was done and not to refer to what was found. All details about the participants should be placed in the Results.

Study details have now been moved to the Methods, and participant information that was previously in the Methods (e.g. number of participants included in the analysis) have now been moved to the start of the Results section.

3. Please explain who in this service is categorised as a health or social care professional. Please explain whether administrative or ancillary staff were included or excluded from this service.

Some further information has been provided in the Participants section of the paper, with reference to another published paper which offers a service mapping of the four Hubs involved in the study (Allsopp et al., 2024).

4. It is not clear what the mental health service comprised. Please provide details of staffing, funding, whether it was a physical facility, the number of sessions available to individuals, what the 'evidence-based' treatments were, whether there was access 24 hours a day to enable shift workers to attend or only office hours, and what NICE Guidance recommends for occupational mental health in this circumstance.

Further information is available in the service mapping paper mentioned above, and the Allsopp et al (2024) paper has now been signposted in this paper, which provides the information above.

5. A description of the funding should be put in the appropriate place at the end of the manuscript, not in the middle of the Introduction.

Funding was mentioned briefly to give context to the paper presenting one component of a mixed methods study. The reference to funding has now been removed from the Introduction.

6. The Procedure needs to be described separately from the measures (better titled data sources), please make these distinct.

Contextual information was included in the Measures section to inform the readers of Hub procedures, such as timing of when Hub clients were invited to complete screening questionnaires, however these were clinical procedures relating to data routinely collected as part of service access and clinical care, rather than research procedures. As such we have moved them to the 'Setting' section where the Hub services were described, rather than the Procedures section. We have renamed the section to Data Sources, as suggested by the reviewer.

7. It is surprising to read that clients were 'required' to complete any information when

earlier it is described as a voluntary self-referral process. Please make it clear whether providing information was a hurdle requirement for receiving care?

The term 'required' was used in the context of timing of questionnaires, rather than whether or not questionnaires were mandatory. Hub clients were invited to completed questionnaires in a format that suited them, but would not have presented a hurdle to receiving care. To avoid confusion we have now reworded this section.

8. The explanation of how missing data were managed needs to be in the Methods not at the beginning of the Results.

Information on missing data has now been moved to a subsection of the Analysis section in Results.

9. If the Hubs were an NHS service it would be expected that most clients were NHS employees – who were the other clients and how were they given access?

The Hubs being NHS services does not necessarily explain the fact that most clients were NHS employees, since all publicly funded mental health services in the UK are NHS services and open to the general public. However, as noted above in the response to Reviewer 1, further information has now been provided about other clients accessing the Hubs, both briefly in the text, and in further detail in a separately published paper which we have now signposted to in the text.

10. What is meant by the term 'caseness' – presumably everyone seeking care at the hubs was categorised as a 'case' for administrative purposes.

The term 'caseness' refers to the state of having met the criteria for clinically significant difficulties on a given questionnaire measure. The definition of caseness for each measure is provided in Table 1. We have now added an additional sentence to the text when introducing Table 1 to further clarify this information.

11. 'emotional concerns' is quite a non-specific descriptor and not helpful for service planners elsewhere seeking to identify people at higher need. Please provide a more specific account of what these were and how they were ascertained.

The analysis utilised routinely collected data outlined by Hubs in their clinical screening. 'Emotional concerns' was a single self-reported question on the clinical screening tool, and here we use it to give an indication of whether or not the client felt they had concerns prior to the pandemic, or if their difficulties were only related to their experiences during the pandemic. This information is outlined in the Data Sources section, and for clarity we have now included an example of the exact question asked of clients ("Were you concerned about your emotional wellbeing/mental health before COVID-19?").

12. As this was a service for mental health support, it is not surprising that clients had scores outside the average ranges. However, no effort is made to compare with data

generated either in the UK or elsewhere about equivalent sub-population or general population data which would assist.

In the discussion section of the manuscript, we have made specific comparisons between our findings and those of other investigations in similar occupational groups / settings, including relevant meta-analyses of this research literature (e.g. Adibi et al. 2022).

13. No effort is made to estimate what fraction of the workforce accessed the hubs, but this is essential in advocating for equivalent services elsewhere. Please add this information.

We cannot estimate precisely these figures due to multiple and diverse occupational groups and health and social care organisations that were eligible for support at different times during the Hub's operational development (i.e. the 'reach' of several Hubs was flexed according to local capacity and commissioning decision over the course of the pandemic) and also lack of precise workforce statistics for all occupational groups that could interface with Hubs (and what proportion of them were in need of support).

14. … between 84% of clients identified as … the other alternative is missing

Apologies – the inclusion of 'between' in this sentence is incorrect and has now been removed. This paragraph is providing a high level overview of the predominant demographic characteristics of the sample. A detailed breakdown of all sample demographics is outlined in full in Table 2.

15. The analyses appeared to be to identify the characteristics of people with the most severe symptoms, but it was not clear what the purpose of doing this was? This information could carry risks for potential employees that those with a history of mental health problems might be considered less employable because deteriorations in mental health and functioning occurred in response to the extra demands of the pandemic. This potential harm of the study should be considered in the Discussion.

Workplace discrimination on the grounds of mental health is, sadly, an enduring phenomenon that far precedes the eventual publication of our findings. While we appreciate that any information pertaining to factors that may predict heightened mental health needs in the health and social workforce may be hypothetically used by unscrupulous employers to discriminate against workers presenting certain characteristics, there are extensive legal protections to prevent such unlawful occurrences in the country where this research was conducted (England, UK), including legislation that forbids enquiry about the mental health history of job applicants, other than circumstances where the applicant can directly benefit from any 'positive action' the employer has taken to improve the employment rate of people with specific characteristics and jobs that require specific individual characteristic (e.g. 'peer mental health workers', a professional role that is becoming more common in many NHS mental health services, must by definition have a personal history of mental health issues and it is therefore lawful to enquire about this at the recruitment stage). The breach of any requirement of such legislation (in particular the Equality Act 2010) is a serious legal matter which can incur severe sanctions for employing organisations and recruitment managers. The enduring reality of workplace discrimination cannot be trivialised or cast-off, but we do not believe that there is a realistic

danger that our research (which ultimately aims to inform the design and planning of more efficient and effective forms of support for the health and social care workforce) will increase risk of discriminatory employment practices.

We have further clarified the overall purpose of the analyses presented in this paper in the final paragraph of the Introduction section:

"As newly established services, the characteristics of people accessing Hub services during the pandemic, or how these characteristics related to mental health need, was not known. Information regarding the characteristics of staff who presented with more severe mental health issues (i.e. potentially requiring more intensive and bespoke mental health support) gathered during the pandemic may be beneficial to the more efficient planning of support services such as the Resilience Hubs should their activation be required in response to novel crises, and/or ongoing staff mental health support initiatives. The objectives of this study were therefore to analyse the demographic and occupational characteristics of health and social care staff who accessed four Resilience Hubs for support during the pandemic, to explore characteristics that were associated with higher mental health need and work and social functional difficulties, to identify health and social care staff who may benefit from mental health support."

> *16. The sample is selected and unrepresentative on several grounds. It could only include people who went to Hubs, and among these, only those who consented. It is well known that service users differ from the general population, and that only limited generalisation can be made. These biases mean that few generalisations can be made to the wider workforce or to other settings and in my opinion the implications should be framed very cautiously and be carefully qualified.*

The Reviewer is correct. However we would like to highlight that 1) Our aim is not to generalise findings to the general populations or the wider workforce, instead 2) The aim of the research (which we have now further clarified) is to inform future health service delivery for similar services that may be set up in the future in response to similar large-scale crises (in fact, this work is funded by the 'Health and Social Care Delivery' programme of the UK's National Institute of Health and Care Research – a specific government-funded research body which aims to support research to improve the quality, accessibility and organisation of health and social care services); 3) We have interpreted all our findings cautiously, without any undue generalisations or attempts to generalise to the wider workforce, but when relevant we have simply highlighted parallel findings that have been observed in other contexts or populations.

> *18. No outcome or economic data which might indicate the impact or cost-effectiveness of these services or consumer-based evaluations are provided. In the absence of these it is difficult to establish the worth of the data to an international readership or to health policymakers and service planners in other settings. In the absence of any prospective or other evaluation data it seems premature and speculative to suggest that these services were beneficial to mental health. These limitations should be considered in the Discussion.*

As mentioned in our manuscript, the analyses presented in this paper are part of a larger multi-component and interdisciplinary programme of research, which included health economic analyses (which are currently being published elsewhere) as well as consumer-based

evaluations in the form of in-depth qualitative interviews with Hub clients, Hub staff and other stakeholders (which have been published in BMJ Open and are already cited in our manuscript – Allsopp et al., 2023). We acknowledge the implications of our programme of research can be fully appreciated and appraised when the findings of these diverse components can be considered together, but the purpose of this journal article is to report a more contained (but already extensive) set of analyses conducted as part of the broader evaluation of the Resilience Hubs.

We further note that 1) our interpretations of the data are already cautious, and nowhere in our manuscript do we make statements regarding the clinical effectiveness of the Hub, 2) we have already highlighted the limitations of the study that preclude the formulation of any conclusion regarding the effectiveness of the Hubs, and made sensible recommendations for future in light of these considerations (i.e. the need for longitudinal and comparative research designs), 3) We have now slightly adjusted the discussion section to further clarify that any statements pertaining to the potential value of the Hubs are grounded on our already published qualitative research, and are more clearly attributed to that parallel stream of work as opposed to arising directly from the current findings.

19. Sex and gender are not interchangeable terms. In this paper it appears that categorisation was done on the basis of sex (woman / man) and this should be corrected throughout.

This is incorrect. The Hub screening questionnaires included items assessing gender (which was assessed using the following options 'woman' 'man' and options that allowed clients to identify in another way (e.g. non-binary; describe in another way), as these latter categories had very small numbers in our study data, we have collapsed these additional categories into "Identified in another way"). These gender categories are in contrast with stricter binary categorisations consistent with biological sex (i.e. male / female), which were not used in the Hubs' screening tools.
